# U–Pb dating for zircons from granitic rocks in southwestern Cambodia

**DOI:** 10.1016/j.heliyon.2023.e19734

**Published:** 2023-09-02

**Authors:** Etsuo Uchida, Shinya Nagano, Sota Niki, Ko Yonezu, Takumi Yokokura, Rathborith Cheng, Takafumi Hirata

**Affiliations:** a Department of Resources and Environmental Engineering, School of Creative Science and Engineering, Waseda University, Ohkubo 3-4-1, Shinjuku, Tokyo, 169-8555, Japan; b Geochemical Research Center, Graduate School of Science, The University of Tokyo, Hongo 7-3-1, Bunkyo, Tokyo, 113-0064, Japan; c Department of Geology, Ministry of Mines and Energy, #79-80, St. 51, Sankat Phsar Thmey III, Khan Daun Penh, Phnom Penh, 12210, Cambodia

**Keywords:** U–Pb dating, Zircon, Granite, Indosinian Orogeny, Mae Ping Fault, Southwestern Cambodia

## Abstract

U–Pb dating was conducted for zircons from a total of 14 samples from 13 granite bodies in southwestern Cambodia using LA-ICP-MS. The granitic rock samples were collected from southwestern Cambodia, southwest of the Mae Ping Fault extending from northwest Cambodia via Tonle Sap Lake to southern Vietnam. The studied rock bodies belong to the ilmenite-series, except for three granitic rock bodies. They were identified as I-or A-type. The analysis yielded three distinct age ranges: 295–309, 191–232, and 75–98 Ma. The 295–309 Ma ages are associated with the Paleo-Tethys Sea subduction beneath the Indochina Block. The ages of 191–232 Ma may correspond to the amalgamation period of the Sibumasu and Indochina Blocks during the Indosinian Orogeny. Granitic rocks with ages of 75–98 Ma occur near the southeastern Cambodia-southern Vietnam border. Formation of these granitic rocks was associated with the Paleo-Pacific Ocean Plate (the Izanagi Plate) subduction beneath the Indochina Block. The region in which these granitic rocks occur is part of the Dalat–Kratie Zone.

## 1 Introduction

**Fig. 1:**
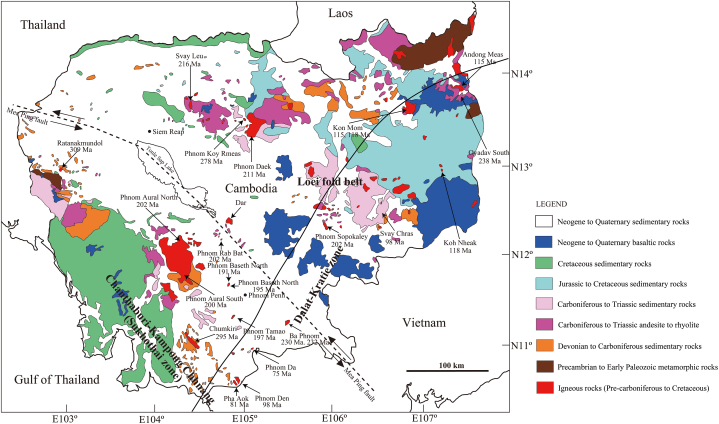
Geologic map of Cambodia [2,3], showing U–Pb ages for zircons separated from igneous rocks in southwestern Cambodia (this study) and northeastern Cambodia [4].

No systematic research has been conducted on the granitic rocks of Cambodia; however, Cheng et al. [1] conducted an initial study of Cambodian granitic rocks, and found that the physical and chemical characteristics of those rocks vary across the Mae Ping Fault, which extends from northwest Cambodia via Tonle Sap Lake to southern Vietnam (Fig. 1) [[2], [3], [4]]. The granitic rocks in the northeastern Cambodia, which is located in northeast of the Mae Ping Fault, are classified as the magnetite-series. They show a strong influence of mantle materials; in contrast, almost all granitic rocks in the southwestern Cambodia belong to the ilmenite-series and show a strong influence of continental crustal materials. Granitic rocks in southwestern Cambodia and southeastern Thailand exhibit similar geochemical signatures and are part of a single unit [5,6]. Cheng et al. [1] determined ages of Cambodian granitic rocks using Rb–Sr dating for the whole-rock. However, the measurement accuracy was not high. Therefore, the measurement accuracy was further improved by Kasahara et al. [4] using a U–Pb dating method for zircons from granitic rocks in northeastern Cambodia. However, except for a few cases, U–Pb dating for zircons separated from granitic rocks in southwestern Cambodia has not been conducted [7,8]. Therefore, U–Pb dating was performed for zircons of granitic rocks collected in southwestern Cambodia in this study. It should be noted that U–Pb dating for zircon particles was previously performed on some granitic bodies of southwestern Cambodia and southeastern Thailand (Chanthaburi zone) by Veeravinantanakul et al. [9] and Uchida et al. [6].

In this paper, we discuss the relationship between the genesis of granitic rocks and tectonics in Cambodia and its neighboring countries on the basis of the results of U–Pb dating for zircons of Cambodian granitic rocks.

## 2 Materials and methods

### 2.1 Materials

U–Pb dating was conducted for zircons separated from 14 samples from 13 granite bodies collected by Cheng et al. [1]. The samples subjected to age dating were as follows: sample CG302 from Phnom Baseth North; sample CG306 from Phnom Baseth South; sample CG308 from Dar; sample CG313 from Phnom Aural North; sample CG316 from Phnom Aural South; sample CG318 from Chumkiri; sample CG323 from Phnom Tamao; sample CG402 (ilmenite-series) and sample CG402 (magnetite-series) from Ba Phnom; sample CG407 from Phnom Da; sample CG409 from Phnom Rab Bat; sample CG411B from Ratanakmundol; sample CG319 from Pha Aok; and sample CG321 from Phnom Den. Locations of studied granitic rock bodies are shown in Fig. 1.

The granitic rock samples were pulverized using an iron mortar and sieved to obtain particles less than 250 μm in diameter. The pulverized samples were subjected to panning in water to remove low-density minerals. After the samples had been dried in an oven, magnetic and non-magnetic minerals were separated from each other by a neodymium magnet. Then non-magnetic minerals were put into a sodium polytungstate solution of a density of about 2.8. Centrifugation was performed under conditions of 3000 rpm for 30 min, and the heavier minerals that settled at the bottom of the container were aspirated using a pipette and collected in a beaker. Next, pure water was added and ultrasonic cleaning was performed, followed by drying. Subsequently, zircon particles were handpicked using a stereomicroscope and embedded in a thinly coated petrographic epoxy resin (PETROPOXY 154, Burnhm Petrographics LLC., ID, USA) on a glass slide. Each epoxy resin on a glass slide containing approximately 20 zircon particles was heated to 140 °C and cured. Then, using #1200 waterproof sandpaper, the zircon particles were polished until the centers were exposed. Further polishing of the sample surface was carried out using 3-μm and then 0.25-μm diamond pastes.

The polished zircon surface was coated with carbon using a Quick Carbon Coater (SC–701C, Sanyu Denshi Co. Ltd., Tokyo, Japan). Subsequently, cathodoluminescence images were captured using a MonoCL3 detector (Gatan, CA, USA) which is attached to a SEM JSM-7001F (JEOL, Tokyo, Japan).

### 2.2 U–Pb dating for zircons using a laser ablation-inductively coupled plasma-mass spectrometry

A laser ablation-inductively coupled plasma-mass spectrometry (LA-ICP-MS) system was used for U–Pb dating. Pre-ablation was conducted on a 40 μm square area using a femtosecond laser ablation system (Cyber Probe UV Plus) to remove contaminants from the zircon surfaces. The pre-ablation was conducted once with a laser output of 30 mW. Subsequently, a laser with a beam diameter of 10 μmφ, an output of 30 mW, a frequency of 2 Hz, and an energy of 3 J/cm^2^, was applied to the center of the pre-ablated region. The integration time was 22 s. The 10–15 μmφ pit was produced by laser ablation. The sample volatilized by laser irradiation was introduced into a Nu Plasma 2 multicollector ICP-MS (Nu Instruments, Wrexham, UK) with a mixed gas of He and Ar at a flow rate of 0.6 l/min. ^206^Pb, ^207^Pb and ^235^U were detected using Daly collectors, ^202^Hg, ^204^(Hg + Pb) and ^208^Pb were detected using secondary electron multipliers, and ^232^Th was detected using a Faraday cup. For detailed information regarding the measurements, refer to Kasahara et al. [4].

The concentration of ^204^Pb was calculated from the following equation:

(1) P204b=Hg+Pb204−H202g×Hg+Pb204H202gblank.

The amount of ^235^U was determined using the following equation [10]:

(2) U235=U238×1137.88.

The measurements were performed by visually examining cathodoluminescence images and selecting areas without inclusions or cracks, and conducting measurements at one location per particle. The NIST SRM 612 glass standard with an ^207^Pb/^206^Pb isotopic ratio of 0.9073 [11] was used to calibrate high-gain ion detectors. In order to calibrate the ^206^Pb/^238^U ratio, the Nancy 91,500 zircon standard with a ^206^Pb/^238^U ratio of 0.17928 ± 0.00018 and a ^207^Pb/^206^Pb ratio of 0.07556 ± 0.00032 was used as a primary standard sample [12]. A GJ-1 zircon standard with an ^206^Pb/^238^U age of 600.4 Ma [13] and an OD-3 zircon standard with an ^206^Pb/^238^U age of 33.0 Ma [14] were used as secondary standards. In one measurement cycle, three points were measured for NIST SRM612, three points for GJ-1, two points for OD-3, a maximum of thirteen points for the studied samples, two points for OD-3 again, three points for NIST SRM612, and three points for GJ-1.

Wetherill diagrams for the concordant zircon samples, except for sample CG407 from Phnom Da, were created using IsoplotR [15], and the formation age was obtained. If the 95% confidence level error ellipse of a zircon particle overlapped with the concordia curve, the zircon particle was determined to be concordia. The concordia age was calculated using a two-dimensionally weighted mean for ^207^Pb/^235^U and ^206^Pb/^238^U ratios [16].

## 3 Geology and sample description

Fig. 1 is a geological map of Cambodia. The Mae Ping Fault could be presumed to run from northwestern Cambodia via Tonle Sap Lake to southern Vietnam. Central Cambodia is occupied by Neogene to Quaternary sediments. In and around the Cardamom Mountains in south westernmost Cambodia, Devonian to Cretaceous sedimentary rocks are widely distributed. Permian to Cretaceous igneous rocks are also observed. In addition, a few amounts of basaltic rocks of Neogene to Quaternary are distributed in the Cardamom Mountains.

**Fig. 2:**
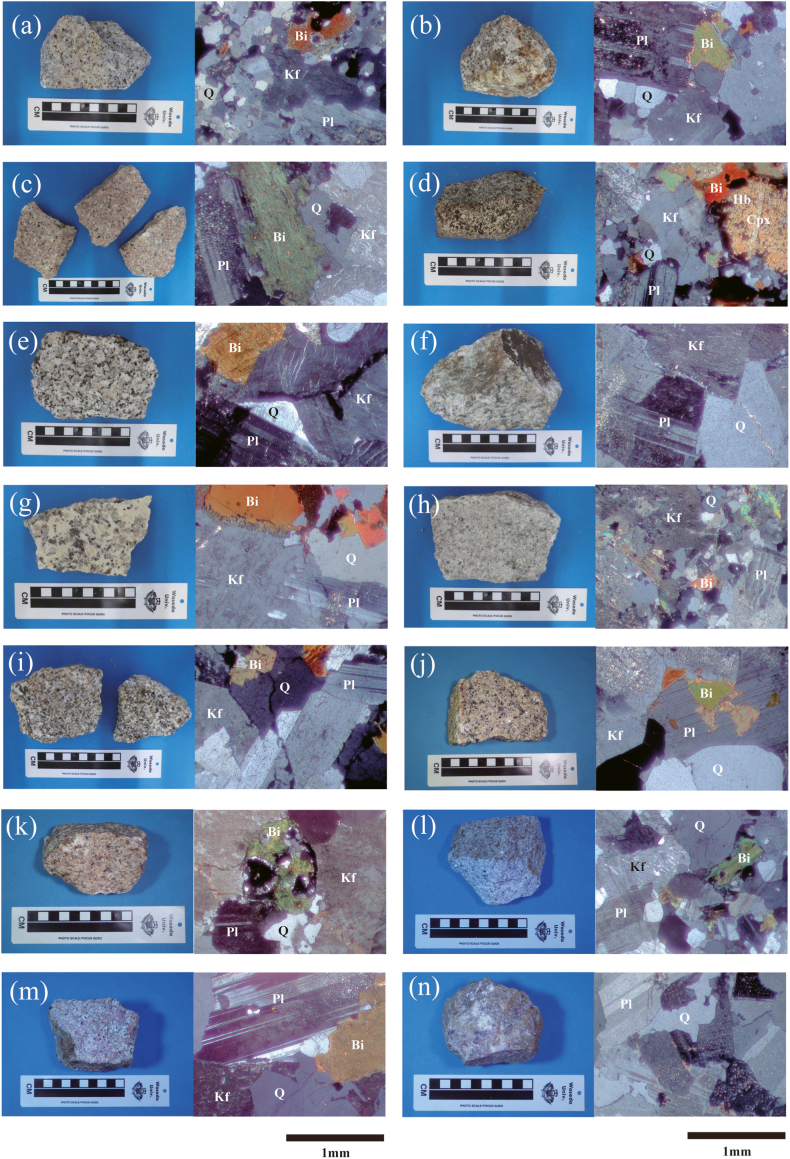
Photographs (left) and photomicrographs under a polarizing microscope (right) of the granitic rocks taken from southwestern Cambodia. (a) Sample CG302 from Phnom Baseth North, (b) sample CG306 from Phnom Baseth South, (c) sample CG308 from Dar, (d) sample CG313 from Phnom Aural North, (e) sample CG316 from Phnom Aural South, (f) sample CG318 from Chumkiri, (g) sample CG323 from Phnom Tamao, (h) sample CG402 (ilmenite series) and (i) sample CG402 (magnetite series) from Ba Phnom, (j) sample CG407 from Phnom Da, (k) sample CG409 from Phnom Rab Bat, (l) sample CG411B from Ratanakmundol, (m) sample CG319 from Pha Aok, and (n) sample CG321 from Phnom Den. Abbreviations: Q, quartz; Pl, plagioclase; Kf, potassium feldspar; Bi, biotite; Hb, hornblende; and Cpx, clinopyroxene.

**Fig. 3:**
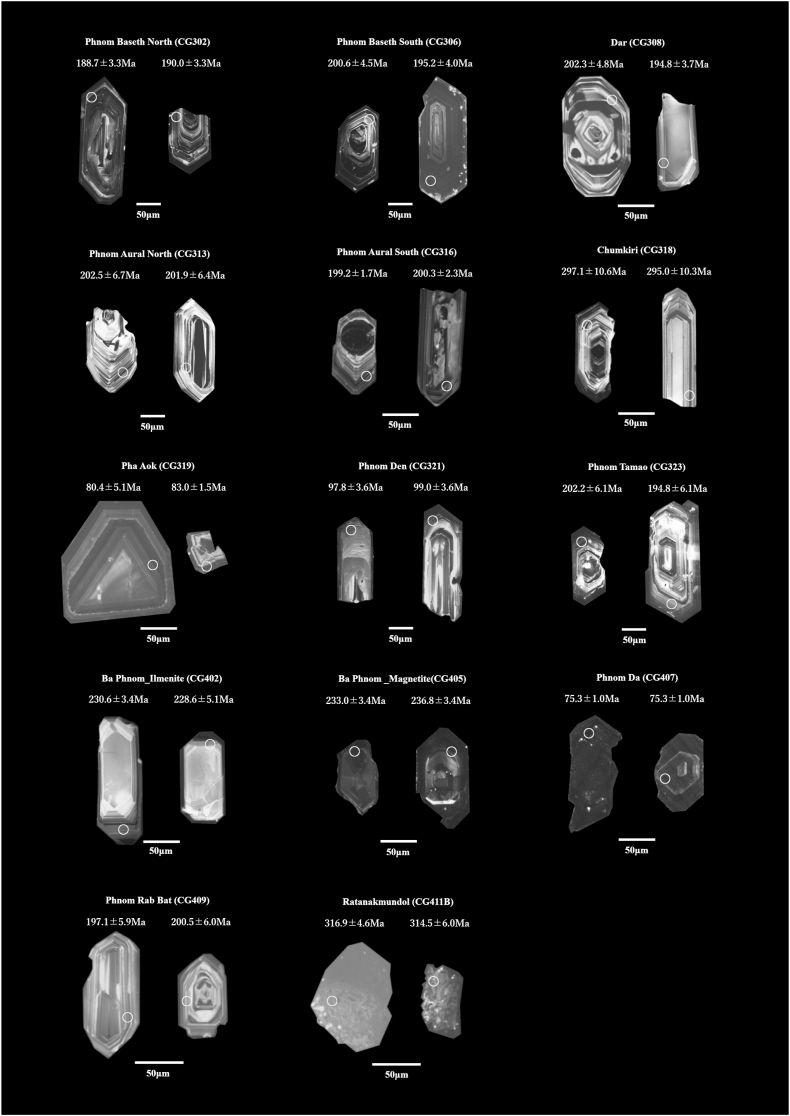
Cathodoluminescence images for zircon gains of granitic rocks from southwestern Cambodia. The analyzed points are circled. (^206^Pb/^238^U) ages are shown in the figure.

Sampling locations and major constituent minerals are summarized in Table 1. Fig. 2 shows photographs and polarized light microscope images of the collected samples. Fig. 3 shows representative cathodoluminescence images of zircon particles.

**Table 1:**
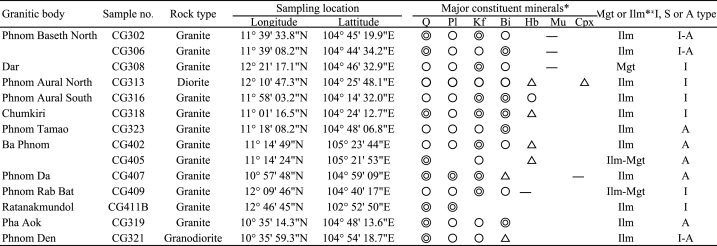
Granitic rock samples conducted for zircon U–Pb dating with sampling locations, major constituent minerals, and rock types.

### 3.1 Sample CG302 from Phnom Baseth North

Major constituent minerals of sample CG302 are quartz, K-feldspar, plagioclase, and biotite (Fig. 2a) [1]. This sample is an ilmenite-series granite classified as I- or A-type.

The zircon particles are pale yellow in color and columnar in shape, with lengths ranging from approximately 80 to 200 μm. Clear zoning patterns were observed in the cathodoluminescence images.

### 3.2 Sample CG306 from Phnom Baseth South

Sample CG306 contains quartz, K-feldspar, plagioclase, and biotite as major constituent minerals (Fig. 2b) [1], and is an ilmenite-series granite classified as I- or A-type.

The zircon particles are columnar to granular in shape, and approximately 90–390 μm long. Clear zoning patterns were observed in the cathodoluminescence images.

### 3.3 Sample CG308 from Dar

Sample CG308 is a granite with magnetic susceptibility indicating a transition between ilmenite-series and magnetite-series, and is classified as I-type. The sample contains K-feldspar, quartz, plagioclase, and biotite as major constituent minerals (Fig. 2c) [1].

The zircon particles are pale yellow, exhibit elongated columnar shapes, and are approximately 60–250 μm long. Clear zoning patterns were observed in the cathodoluminescence images.

### 3.4 Sample CG313 from Phnom Aural North

Sample CG313 is an ilmenite-series diorite classified as I-type. This sample consists mainly of quartz, K-feldspar, plagioclase, biotite, hornblende, and clinopyroxene (Fig. 2d) [1].

The zircon particles are pale yellow and exhibit short columnar to granular shapes, with lengths ranging from approximately 60 to 220 μm. Clear zoning patterns were observed in the cathodoluminescence images.

### 3.5 Sample CG316 from Phnom Aural South

Major constituent minerals of sample CG316 are K-feldspar, quartz, plagioclase, and biotite (Fig. 2e) [1]. This sample is an ilmenite-series granite classified as I-type.

The zircon particles are elongated columnar to short columnar in shape, and approximately 80–300 μm long. Clear zoning patterns were observed in the cathodoluminescence images.

### 3.6 Sample CG318 from Chumkiri

Sample CG318 is an ilmenite-series granite classified as I-type, and contains quartz, plagioclase, K-feldspar, and biotite as major constituent minerals (Fig. 2f) [1].

The zircon particles are pale yellow and exhibit elongated columnar to short columnar shapes, with lengths ranging from approximately 100 to 300 μm. Clear zoning patterns were observed in the cathodoluminescence images.

### 3.7 Sample CG323 from Phnom Tamao

Sample CG323 consists quartz, plagioclase, K-feldspar, and biotite (Fig. 2g) [1]. It is an ilmenite-series granite classified as A-type.

The zircon particles are pale yellow, columnar in shape, and approximately 80–300 μm long. Clear zoning patterns were observed in the cathodoluminescence images.

### 3.8 Samples CG402 (ilmenite-series) and CG405 (magnetite-series) from Ba Phnom

Major constituent minerals of sample CG402 are K-feldspar, quartz, plagioclase, biotite, and hornblende (Fig. 2h) [1]. This sample is an ilmenite-series granite classified as A-type. Sample CG405 is an ilmenite-to magnetite-series granite classified as A-type, and contains K-feldspar, quartz, plagioclase, biotite, and hornblende as the main rock-forming minerals (Fig. 2i) [1].

The zircon particles in CG402 are pale yellow, columnar, and approximately 100–200 μm long. Clear zoning patterns were observed in the cathodoluminescence images. The zircon particles in CG405 are columnar and approximately 70–150 μm long. Clear zoning patterns were observed in the cathodoluminescence images.

### 3.9 Sample CG407 from Phnom Da

Sample CG407 is an ilmenite-series granite classified as A-type. The sample contains quartz, plagioclase, K-feldspar, and biotite as major constituent minerals (Fig. 2j) [1].

The zircon particles are pale yellow to gray in color, exhibit short columnar shapes, and range from approximately 100 to 150 μm in length. Zoning patterns were observed in the cathodoluminescence images. The uranium concentration in the zircons is exceptionally high, exceeding 10,000 ppm.

### 3.10 Sample CG409 from Phnom Rab Bat

Major constituent minerals of sample CG409 are K-feldspar, quartz, plagioclase, and biotite (Fig. 2k) [1]. This sample is an ilmenite- to magnetite-series granite classified as I-type.

The zircon particles are pale yellow in color, elongated columnar to short columnar in shape, and approximately 60–200 μm long. Distinct zoning patterns were observed in the cathodoluminescence images.

### 3.11 Sample CG411B from Ratanakmundol

Sample CG411B is an ilmenite-series granite classified as I-type. The main rock-forming minerals are quartz and plagioclase (Fig. 2l) [1].

The zircon particles are approximately 40–150 μm long and short columnar to granular in shape. Clear zoning patterns were observed in the cathodoluminescence images.

### 3.12 Sample CG319 from Pha Aok

Sample CG319 is an ilmenite-series granite classified as A-type, and contains quartz, plagioclase, K-feldspar, and biotite as major constituent minerals (Fig. 2m) [1].

The zircon particles are short columnar to granular in shape, and ca. 50–300 μm long. Clear zoning patterns were observed in the cathodoluminescence images.

### 3.13 Sample CG321 from Phnom De

Major constituent minerals of sample CG321 are quartz, plagioclase, K-feldspar, biotite, and muscovite (Fig. 2n) [1]. This sample is an ilmenite-series granodiorite classified as I- or A-type.

The zircon particles are long columnar to granular in shape, and approximately 100–300 μm long. Zoning patterns were observed in the cathodoluminescence images.

## 4 Zircon U–Pb dating results

**Fig. 4:**
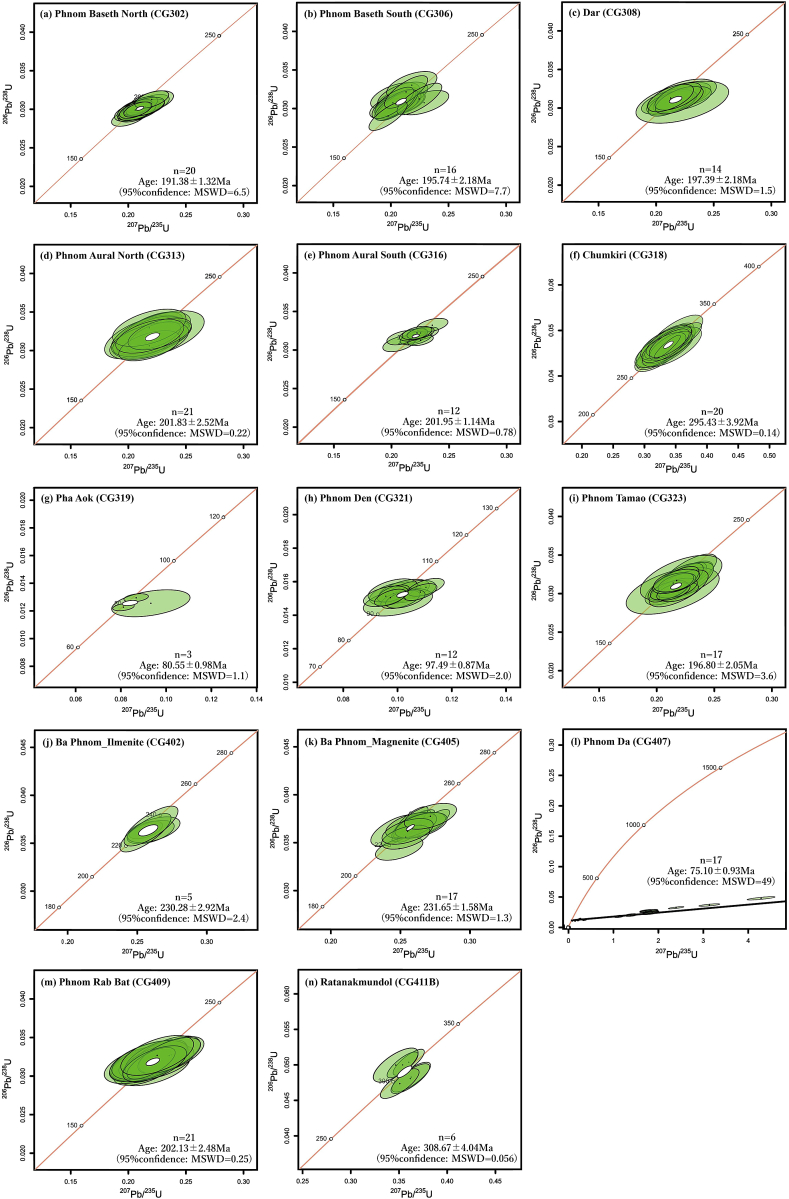
Concordia and discordia (CG407) diagrams for zircon particles separated from granitic rocks in southwestern Cambodia. (a) Sample CG302 from Phnom Baseth North, (b) sample CG306 from Phnom Baseth South, (c) sample CG308 from Dar, (d) sample CG313 from Phnom Aural North, (e) sample CG316 from Phnom Aural South, (f) sample CG318 from Chumkiri, (g) sample CG323 from Phnom Tamao, (h) sample CG402 (ilmenite series) and (i) sample CG402 (magnetite series) from Ba Phnom, (j) sample CG407 from Phnom Da, (k) sample CG409 from Phnom Rab Bat, (l) sample CG411B from Ratanakmundol, (m) sample CG319 from Pha Aok, and (n) sample CG321 from Phnom Den. Abbreviation: MSWD, mean square weighted deviation.

The U–Pb dating results are shown in Fig. 4 and also summarized in Supplementary Table S1.

### 4.1 Sample CG302 from Phnom Baseth North

U–Pb dating was performed for 25 zircon particles. Among them, 20 particles yielded concordant data. As a result, the concordia age of 191.38 ± 0.34 Ma was obtained (Fig. 4a).

### 4.2 Sample CG306 from Phnom Baseth South

U–Pb dating was carried out for 32 zircon particles. Among them, 17 particles produced concordant data. As a result, the concordia age of 195.04 ± 1.01 Ma was obtained (Fig. 4b).

### 4.3 Sample CG308 from Dar

U–Pb dating was conducted for 25 zircon particles, of which 14 particles yielded concordant data. As a result, the concordia age of 197.39 ± 0.56 Ma was obtained (Fig. 4c).

### 4.4 Sample CG313 from Phnom Aural North

U–Pb dating was performed for 24 zircon particles, and concordant data were obtained from 21 particles. As a result, the obtained concordia age is 201.83 ± 0.64 Ma (Fig. 4d).

### 4.5 Sample CG316 from Phnom Aural South

U–Pb dating was conducted for 20 zircon particles. of which 14 particles yielded concordant data. As a result, the concordia age of 200.37 ± 0.23 Ma was obtained (Fig. 4e).

### 4.6 Sample CG318 from Chumkiri

U–Pb dating was conducted for 25 zircon particles, and concordant data were obtained from 20 particles. As a result, the concordia age of 295.43 ± 1.00 Ma was obtained (Fig. 4f).

### 4.7 Sample CG323 from Phnom Tamao

U–Pb dating was conducted for 24 zircon particles, of which 16 particles produced concordant data. As a result, the concordia age of 197.41 ± 0.55 Ma was obtained (Fig. 4g).

### 4.8 Samples CG402 (ilmenite-series) and CG405 (magnetite-series) from Ba Phnom

For sample CG402, U–Pb dating was carried out for 24 zircon particles. Concordant data were obtained from 5 particles. As a result, the concordia age of 230.28 ± 0.74 Ma was obtained (Fig. 4h).

For sample CG405, U–Pb dating was conducted for 21 zircon particles. Among them, 17 particles produced concordant data. As a result, the concordia age of 232.14 ± 0.72 Ma was obtained (Fig. 4i).

### 4.9 Sample CG407 from Phnom Da

U–Pb dating was conducted for 18 zircon particles. However, only one particle fell on the concordia curve; therefore, a discordia age was determined. The obtained discordia age is 75.09 ± 0.24 Ma (Fig. 4j).

### 4.10 Sample CG409 from Phnom Rab Bat

U–Pb dating was conducted for 26 zircon particles. Among them, 21 particles fell on the concordia curve. As a result, the concordia age of 202.13 ± 0.63 Ma was obtained (Fig. 4k).

### 4.11 Sample CG411B from Ratanakmundol

U–Pb dating was conducted for 15 zircon particles, of which 6 particles fell on the concordia curve. As a result, the concordia age of 309.12 ± 1.83 Ma was obtained (Fig. 4l).

### 4.12 Sample CG319 from Pha Aok

U–Pb dating was conducted for 7 zircon particles. Among them, 3 particles fell on the concordia curve. As a result, the concordia age of 80.89 ± 0.95 Ma was obtained (Fig. 4m).

### 4.13 Sample CG321 from Phnom Den

U–Pb dating was conducted for 16 zircon particles, of which 12 particles fell on the concordia curve. As a result, the concordia age of 97.72 ± 0.79 Ma was obtained (Fig. 4n).

## 5 Discussion

### 5.1 Stages of magma activity in Cambodia

U–Pb dating was previously conducted on only three granitic bodies, namely Phnom Tamao, Pha Aok, and Phnom Den, located in southwestern Cambodia (Chanthaburi–Kampong Chhnang zone or Sukhothai zone) by Nong et al. [7,8], and produced ages of 187.8 ± 3.2, 78.8 ± 1.1, and 93.5 ± 1.5 Ma, respectively. The ages obtained in this study are in close agreement, with ages of 197.41 ± 0.55 Ma for Phnom Tamao, 80.89 ± 0.95 Ma for Pha Aok, and 97.72 ± 0.79 Ma for Phnom Den.

The U–Pb ages for zircon grains of the granitic rocks from southwestern Cambodia can be divided into three main ranges: 295–309, 191–232, and 75–98 Ma. When U–Pb dating results of the granitic rocks taken from northeastern Cambodia is included, age ranges extend only slightly to 278–309, 191–238, and 75–118 Ma, respectively [4].

The 278–309 Ma age range has been hypothesized to be associated with the Paleo-Tethys Sea subduction beneath the Indochina Block [18,19].

Many granitic rocks in Cambodia, including those in northeastern Cambodia, have ages between 191 and 238 Ma. This period corresponds to a time of the amalgamation period of the Sibumasu and Indochina Blocks during the Indosinian Orogeny [4,17,[20], [21], [22], [23]]. Similar ages were also obtained for granitic rocks taken from Chanthaburi and Chachoengsao provinces of southeastern Thailand [6]. These provinces could be considered an extension of southwestern Cambodia.

Granitic rocks with ages between 75 and 118 Ma occur near the border between southeastern Cambodia and southern Vietnam, and are distinct from the rocks of northeastern and southwestern Cambodia. These younger-aged granitic rocks formed in association with the Paleo-Pacific Ocean Plate (the Izanagi Plate) subduction beneath the Indochina Block [24]. The region where these younger-aged granitic rocks are distributed is called the Dalat–Kratie zone [25].

### 5.2 Nd–Sr isotope

**Fig. 5:**
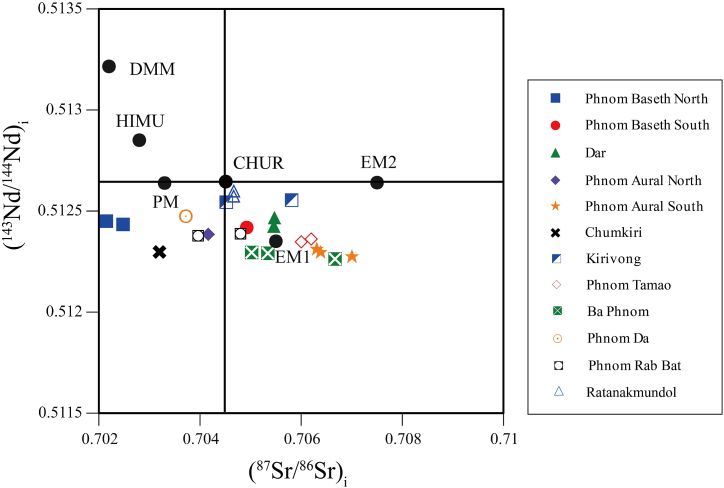
Diagram for (^143^Nd/^144^Nd)_i_ vs. (^87^Sr/^86^Sr)_i_ initial values of the studied granitic rocks. Abbreviations: PM, primitive mantle; CHUR, chondritic uniform reservoir; DMM, depleted mid-ocean-ridge-basalt mantle; HIMU, high μ; EM1, enriched mantle 1; and EM2, enriched mantle 2. The data of (^143^Nd/^144^Nd)_i_ and (^87^Sr/^86^Sr)_i_ initial values for PM, CHUR, DMM, HIMU, EM1, and EM2 are taken from Faure and Mensing [17] and Schaefer [18].

Cheng et al. [1] determined the isotope ratios of ^143^Nd/^144^Nd and ^87^Sr/^86^Sr for granitic rocks from southwestern Cambodia. U–Pb age values were obtained for each rock body in southwestern Cambodia in this research. (^143^Nd/^144^Nd)_i_ and (^87^Sr/^86^Sr)_i_ initial values were calculated using the obtained ages and a diagram of (^143^Nd/^144^Nd)_i_ vs. (^87^Sr/^86^Sr)_i_ was created (Fig. 5) [17,26]. The (^143^Nd/^144^Nd)_i_ initial values were from 0.5122 to 0.5126 with a narrow range. In contrast, the (^87^Sr/^86^Sr)_i_ initial values were relatively high with a wide range of 0.702–0.707. These values suggest the involvement of continental crustal materials to the granitic rocks in southwestern Cambodia [27]. The obtained initial values are consistent with those of granitic rocks in southeastern Thailand [4], which could be considered an extension of southwestern Cambodia [6].

## 6 Conclusions

U–Pb dating for zircons was conducted on granitic rock samples collected from 13 rock bodies in southwestern Cambodia in this study. The main findings are as follows.

(1) U–Pb concordia ages were determined for 13 samples from 12 granitic rock bodies in southwestern Cambodia. For sample CG407 from the Phnom Da granitic rock body, a discordia age of 75.27 Ma was obtained.
(2) Based on the U–Pb ages using zircon grains, the granitic rocks from both southwestern and northeastern Cambodia were classified into three groups: 278–309, 191–238, and 75–118 Ma.
(3) The 278–309 Ma ages are associated with the Paleo-Tethys Sea Plate beneath the Indochina Block. The 191–238 Ma ages may correspond to the amalgamation period of the Sibumasu and Indochina Blocks. The granitic rocks of the ages of 75–118 Ma were generated with the Paleo-Pacific Plate (the Izanagi Plate) subduction beneath the Indochina Block.

## Author contribution statement

Etsuo Uchida: Conceived and designed the experiments; Analyzed and interpreted the data; Wrote the paper.

Shinya Nagano; Ko Yonezu; Takumi Yokokura: Performed the experiments; Analyzed and interpreted the data.

Sota Niki: Performed the experiments; Contributed reagents, materials, analysis tools or data.

Rathborith Cheng; Takafumi Hirata: Contributed reagents, materials, analysis tools or data.

## Data availability statement

Data included in article/supp. material/referenced in article.

## Declaration of competing interest

The authors declare that they have no known competing financial interests or personal relationships that could have appeared to influence the work reported in this paper.
